# Supplementary material for: Novel Driver Strength Index highlights important cancer genes in TCGA PanCanAtlas patients
Source: PeerJ. 2022 Aug 11;10:e13860. doi: 10.7717/peerj.13860 (PMC9375969; doi:10.7717/peerj.13860)
Supplement: Supplemental Information 2 [file peerj-10-13860-s002.zip › COHORTS/genes plots/detailed/2021_8_16_14_9_distribution_events_detailed_males_16.pdf]

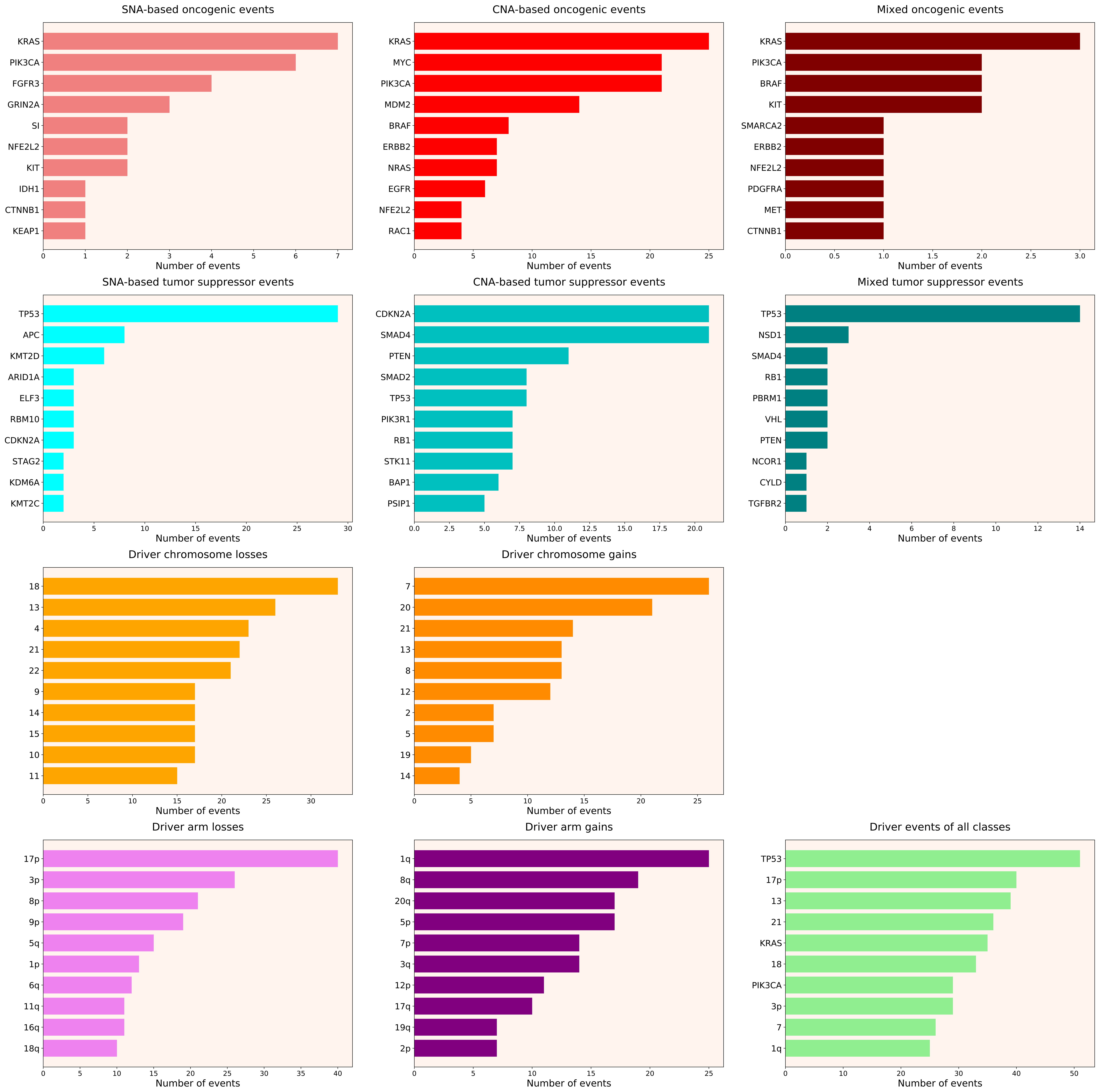

SNA-based oncogenic events
KRAS
PIK3CA
FGFR3
GRIN2A
SI
NFE2L2
KIT
IDH1
CTNNB1
KEAP1
Number of events
CNA-based oncogenic events
KRAS
MYC
PIK3CA
MDM2
BRAF
ERBB2
NRAS
EGFR
NFE2L2
RAC1
Number of events
Mixed oncogenic events
KRAS
PIK3CA
BRAF
KIT
SMARCA2
ERBB2
NFE2L2
PDGFRA
MET
CTNNB1
Number of events
SNA-based tumor suppressor events
TP53
APC
KMT2D
ARID1A
ELF3
RBM10
CDKN2A
STAG2
KDM6A
KMT2C
Number of events
CNA-based tumor suppressor events
CDKN2A
SMAD4
PTEN
SMAD2
TP53
PIK3R1
RB1
STK11
BAP1
PSIP1
Number of events
Mixed tumor suppressor events
TP53
NSD1
SMAD4
RB1
PBRM1
VHL
PTEN
NCOR1
CYLD
TGFBR2
Number of events
Driver chromosome losses
18
13
4
21
22
9
14
15
10
11
Number of events
Driver chromosome gains
7
20
21
13
8
12
2
5
19
14
Number of events
Driver arm losses
17p
3p
8p
9p
5q
1p
6q
11q
16q
18q
Number of events
Driver arm gains
1q
8q
20q
5p
7p
3q
12p
17q
19q
2p
Number of events
Driver events of all classes
TP53
17p
13
21
KRAS
18
PIK3CA
3p
7
1q
Number of events
